# National HIV Testing Day — June 27, 2013

**Published:** 2013-06-21

National HIV Testing Day, June 27, promotes the importance of testing in detecting, treating, and preventing human immunodeficiency virus (HIV) infection. HIV testing is the essential entry point to a continuum of prevention, health-care, and social services that improve the quality of life and the length of survival for persons with HIV (1). Persons with HIV who receive appropriate treatment, monitoring, and health care also reduce their chances of transmitting HIV to others. CDC recommends that all persons aged 13–64 years be screened for HIV in health-care settings located in areas where the prevalence of undiagnosed HIV infection is >0.1%, and that persons with increased risk for HIV be retested at least annually (2).

In April 2013, the U.S. Preventive Services Task Force updated its 2005 guidelines on HIV screening, to recommend that clinicians screen all persons aged 15–65 years for HIV infection at least once, regardless of their risk; that younger adolescents and older adults with increased risk also be screened; and that persons with increased risk be screened more frequently (3). These updated recommendations are based on increasing evidence of the benefits of early antiretroviral therapy for HIV-infected persons and its effectiveness in preventing HIV transmission. Additional information is available at http://www.uspreventiveservicestaskforce.org/uspstf13/hiv/hivfinalrs.htm#summary, http://www.cdc.gov/features/hivtesting, and http://www.hivtest.cdc.gov.
